# Differential time allocation of foraging workers in the subterranean termite

**DOI:** 10.1186/s12983-021-00446-5

**Published:** 2021-12-13

**Authors:** Sang-Bin Lee, Thomas Chouvenc, Nan-Yao Su

**Affiliations:** Department of Entomology and Nematology, Ft. Lauderdale Research and Education Center, University of Florida, 3205 College Avenue, Ft. Lauderdale, FL 33314 USA

**Keywords:** Social insect, *C. formosanus*, Foraging behavior, Task division, Task allocation

## Abstract

**Background:**

Foraging in group living animals such as social insects, is collectively performed by individuals. However, our understanding on foraging behavior of subterranean termites is extremely limited, as the process of foraging in the field is mostly concealed. Because of this limitation, foraging behaviors of subterranean termites were indirectly investigated in the laboratory through tunnel geometry analysis and observations on tunneling behaviors. In this study, we tracked subsets of foraging workers from juvenile colonies of *Coptotermes formosanus* (2-yr-old) to describe general foraging behavioral sequences and to find how foraging workers allocate time between the foraging site (food acquisition or processing) and non-foraging site (food transportation).

**Results:**

Once workers entered into the foraging site, they spent, on average, a significantly longer time at the foraging site than the non-foraging site. Our clustering analysis revealed two different types of foraging workers in the subterranean termite based on the duration of time they spent at the foraging site and their foraging frequency. After entering the foraging site, some workers (cluster 1) immediately initiated masticating wood fragments, which they transferred as food boluses to recipient workers at the foraging site. Conversely, the recipient workers (cluster 2) moved around after entering the foraging site and received food from donating workers.

**Conclusions:**

This study provides evidence of task specialization within foraging cohorts in subterranean termites.

**Supplementary Information:**

The online version contains supplementary material available at 10.1186/s12983-021-00446-5.

## Introduction

Animals search for and consume food resources to survive and successfully reproduce, as food acquisition fundamentally supports their development and reproduction. Depending on the lifestyle of a species, foraging can be performed alone (i.e., solitary animals) or in groups, which often requires a collective coordination of actions to optimize foraging output [1]. Among group-living organisms, eusocial insects such as ants, bees, wasps, and termites take task coordination to a different level of complexity, as the reproductive division of labor in these societies results in individuals specialized in tasks other than reproduction, which enabled their evolutionary success in various ecosystem [1–3].

In social insect colonies, foraging is a collective process in which individual workers venture away from the safety of the nest, search for and collect food resources, and return to the nest to provision their nestmates with food. In many cases, foraging is performed by a subset of colony members, indicating that not all individuals in a colony equally participate in foraging [3–5]. The portion of active coordinating foragers is usually context and species-dependent, and it can be challenging to determine foraging behaviors at the colony level because of the large spatial scale in social insect colonies. Therefore, investigations of task allocation processes involved in foraging behavior have historically tracked subsets of foraging workers, as a proxy to infer the theoretical framework behind colony-wide foraging behaviors in social insects [6–10].

Although foraging behavior has extensively been studied in many social insects, most empirical data were obtained from species with readily visible access to foragers such as ants and bees [11–15]. In comparison, studies on foraging behaviors of termites, such as *Coptotermes,* have been largely neglected, as it is difficult to study termites in the field due their large colony size (> one million individuals), unspecified nest location (i.e., underground or inside trees), long foraging distance, and invisible foraging pathways (i.e., foraging via extensive and lengthy underground tunnels and aboveground shelter tubes) [13, 16–19]. Because of their cryptic habit, studies on foraging behavior of subterranean termites have instead primarily focused on tunneling behaviors observed in laboratory arenas [20–26] and tunnel geometry analysis [27–30]. Despite previous studies enabled us to understand food finding process of subterranean termites, foraging behaviors after food discovery are barely understood.

In subterranean termites, foraging is always initiated with the excavation of new underground tunnels toward putative foraging sites [19]. Tunneling behavior in subterranean termites is composed of two components, excavation and deposition [24, 26, 31], and it was shown that subterranean termites do not equally participate in excavations, indicating task allocation among workers during tunnel excavation [20, 22, 23, 31]. However, it remains unknown if task allocation persists after a food item is discovered through excavation, as additional individuals are recruited to the discovered wood resource [32]. Here, we hypothesize that once workers start foraging, not all individuals allocate their time identically. We aimed to investigate if discrete behavioral categories of foragers could be identified.

In this study, subsets of *Coptotermes formosanus* Shiraki (Rhinotermitidae) workers from whole juvenile colonies (2 yr-old) were tracked at a foraging site. We determined how foragers allocate their time performing various behaviors involved in food acquisition or processing (i.e., time spent at the foraging site) and food transportation away from the foraging site (i.e., time spent at the non-foraging site) during a 12 h period. We also measured foraging frequencies of workers to find out how workers persistently participate in foraging tasks to determine if time allocation in foraging behaviors is homogenous among all foraging workers, or if workers would display distinct behavioral profiles.

## Materials and methods

### Colony establishment

Colonies of *C. formosanus* were established using alate (winged primary reproductives) pairs collected during dispersal flights (April–May 2017) following the method used by Chouvenc et al. [33]. Hundreds of alates were collected in Broward County (Florida, USA) using a light trap made of an LED light (1720 lumens), a florescent dark light and a regular light. Collected alates were kept in a container with moist corrugated cardboard and brought back to the laboratory immediately. The sex of the alates was determined morphologically when they dealated. Rearing units consisted of moistened organic soil at the bottom (3 cm high), four pieces of wood (5 × 0.5 × 0.5 cm, *Picea* sp.) on top of the soil, and 3% agar solution poured into a plastic vial (8 cm height × 2.5 cm diameter cm, IntraPac, Plattsburgh, New York, USA). Once the agar solution solidified, a pair of male and female reproductives was introduced and the rearing unit was closed with a perforated cap to allow air circulation. Hundreds of rearing units were prepared and kept at 28 ± 1 ℃. Colonies were processed 6 to 8 months after colony establishment, and surviving colonies were transferred to larger vials (6.3 × 4.6 cm height × diameter, IntraPac, Plattsburgh, NY, USA), containing organic soil (5 cm high), 6 pieces of wood (5 × 0.5 × 0.5 cm, *Picea* sp.), and 3% agar solution.

One year after colony foundation, thriving colonies were transferred to container boxes (1.5 L, 17 × 12 × 7 cm, Pioneer Plastics, Dixon, Kentucky, USA). In these containers, moistened organic soil was placed at the bottom (3–4 cm high) and a piece of wood (14.5 × 4 × 1 cm, *Picea* sp.) was placed on top of the soil. Then, the vial that a colony was reared in for a year was uncapped and horizontally placed on the bottom of the container, allowing termites to forage out. Colonies were monitored weekly with replenishment of wood and water as needed until they were two years old, resulting in colonies with 3000 to 5000 individuals.

### Foraging behavior assay

**Fig. 1:**
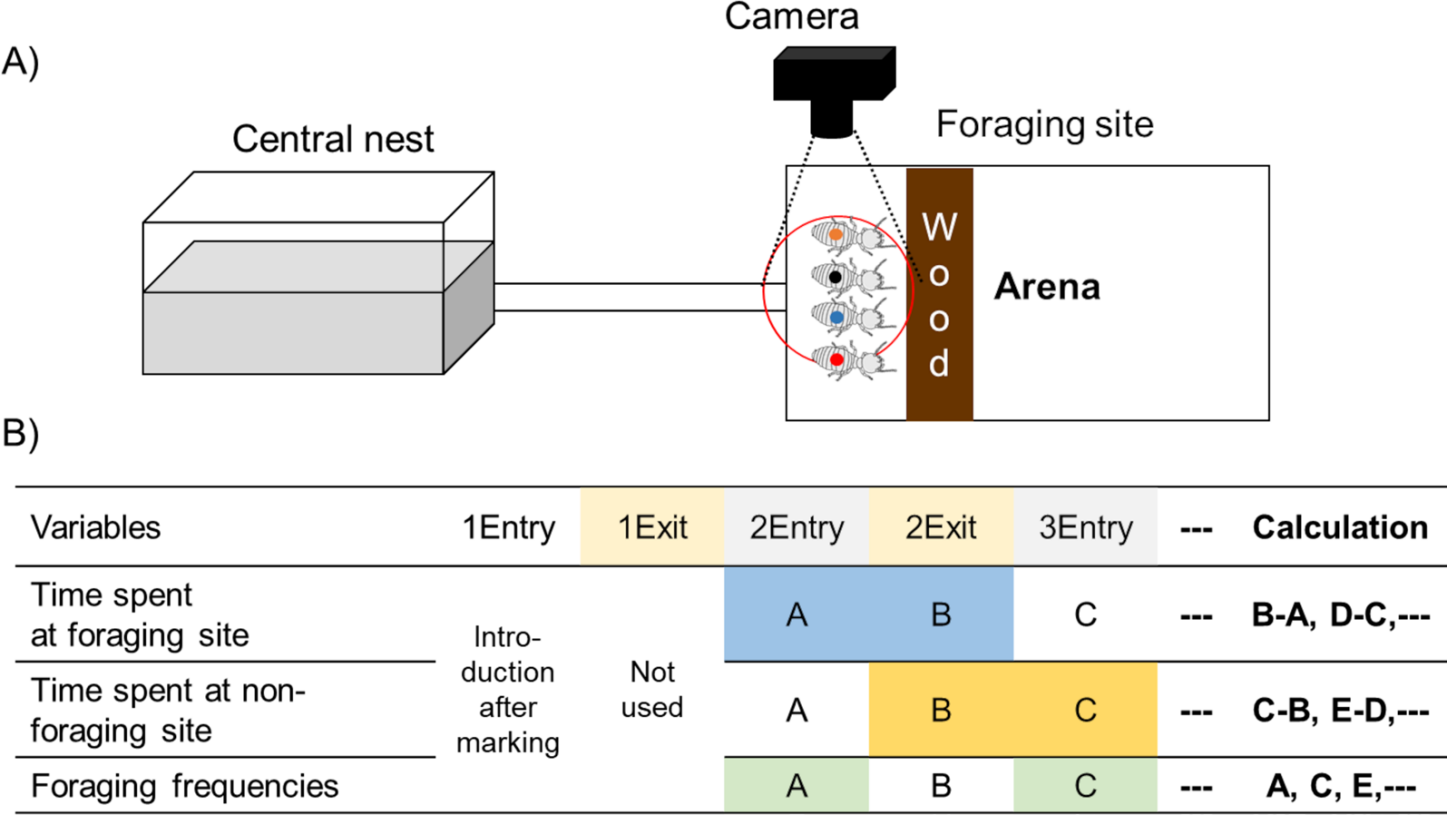
**A** The experimental set-up. The container (left) box was defined as the central nest and the planar arena was used as the foraging site (right). The container and the arena were connected by 1 m length of polygon tube. A camcorder was mounted on top of entry point into the arena, which allowed observation of termites from the piece of wood to the entry point. **B** Examples of variable and how each variable was calculated. After introduction of marked workers into the foraging site, the first exit time was excluded from the data and the time until the next entry was measured

Two years after colony foundation, three visibly healthy colonies were selected for the experiment. A small hole (diameter: 0.5 cm) was drilled on the side of the container to connect it to a planar arena with an acryl tube (length: 1 m, diameter: 0.7 cm) for observation (Fig. 1A). For each colony, the container and arena were defined as the central nest and foraging site, respectively. In the central nest, a reproductive excluder was applied to prevent the reproductives from moving out of the central nest [34] and the piece of wood was removed from the central nest. Then, a piece of wood (*Picea* sp., 14.5 cm × 4 cm × 0.2 cm^3^), soaked in water for 2 days prior to experiment, was placed in the arena (20 × 25 × 0.5 cm^3^) as food. Workers and soldiers were allowed to freely move between foraging site and central nest through the 1 m acryl tube for 3 days. For each colony, 13 termites were randomly collected at the foraging arena near the wood as a subsample of the active foraging population. These individuals were marked with different paint colors on the dorsal surface of their abdomen and re-introduced into the foraging arena. A camcorder (4 HG10 AVCHD High Definition, Canon Inc., Tokyo, Japan) was mounted on top of the entry point of the arena, and video was recorded for 12 h.

Entry and exit times of marked termites at the foraging site entrance were recorded to calculate three parameters: (1) time spent at the foraging site for food acquisition, (2) time spent at the non-foraging site for food transportation or other non-foraging tasks, and (3) foraging frequencies (number of entry events in twelve hours) (Fig. 1B). In all calculations, the first time each individual exited after introduction to the foraging arena was not used for analysis because of possible disturbance during the introduction process.

### Description of foraging worker’s behavioral repertoire

Behavioral repertoire of workers at the foraging site was determined from the videos. Behaviors displayed by the marked termites were recorded from the moment they entered into the foraging site until they exited toward the tunnel. Although duration and frequency of each behavior was not quantified, we were able to determine overall behavioral sequences of foraging workers. In general, six different behaviors of workers at the foraging site were identified: wood fragment collection, wandering, mastication of wood fragments to form a bolus, searching for recipients, receiving food from other termites, and food transfer by “stomodeal trophallaxis”. Workers also exhibited proctodeal trophallaxis at the foraging site. However, we chose to not include this behavior in the behavioral sequence since it was difficult to determine where the food used in the proctodeal trophallaxis comes from.

### Statistical analysis

We calculated the mean value of all incidences of each variable (i.e., time spent at the foraging site and non-foraging site) since each individual worker had multiple observations. This allowed us to have a single value per termite for each variable. Then, average time spent was determined using this individual mean value. Average time spent during the observation period was compared with a Mann–Whitney U test. Comparisons were performed with pooled data and also separately for each colony to confirm variation among colonies.

Next, three variables (time spent at the foraging site, time spent at the non-foraging site and foraging frequency) were further analyzed with K-means clustering analysis in R v3.6.1 and library of tidyverse, cluster and factoextra were used for the clustering analysis [35]. The number of clusters was determined following by the elbow method to calculate total within the sum of squares. For the clustering analysis, three individuals were excluded because they stopped appearing at the foraging site shortly after their re-introduction (individuals #4, #24 and #29). Based on the results of the cluster analysis, two different groups were found, and comparisons between the two different clusters were performed with Mann–Whitney U test.

The proportion of time spent at the foraging site and non-foraging site was calculated for the 12 h observation period and compared between clusters with Wilcoxson’s sum rank test and Fisher’s exact test. All statistical analyses except the clustering analysis were performed under SPSS V19.0 [36]

## Results

### Behavioral repertoire and sequence of foraging workers

**Fig. 2:**
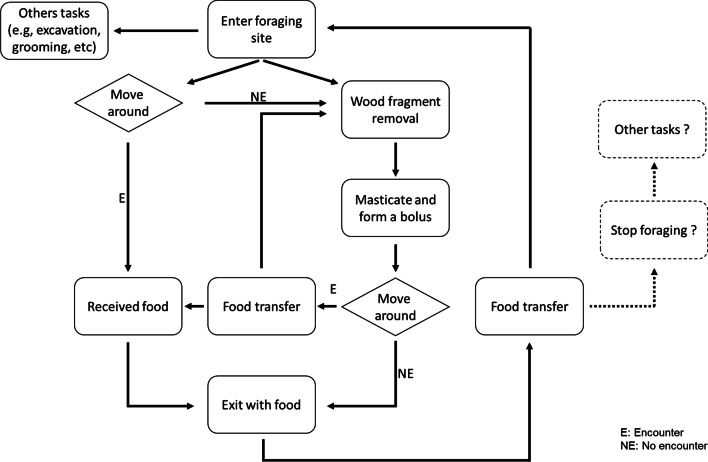
Behavioral sequences of foraging workers in *C. formosanus* at the foraging site. Solid and dashed lines indicate observed and speculated behaviors respectively

After workers entered the foraging site, they either directly moved to the wood piece to remove wood fragments or moved around the wood piece (Fig. 2). Workers that directly headed to the wood piece initiated wood processing by tearing wood particles from accessible wood sections. They then masticated the wood particles to form a bolus. After that, workers often moved around the wood piece instead of immediately transporting food back to the nest after processing. When they encountered any potential recipients near the wood piece, workers transferred the food bolus through stomodeal trophallaxis and then the donor termites returned to the void spaces on the piece of wood. Some workers did not initiate wood processing after entering the foraging site. Instead, they moved around the foraging site until encountering an individual with a food bolus, received food, and then exited from the foraging site.

### Time allocation of workers at the foraging and non-foraging sites

During the observation period, time spent at the foraging site and at the non-foraging site varied depending on the individual (Additional file 1: Table 1). For example, termite IDs #15 and #28 stayed mostly at the foraging site throughout the observation period, while #9 and #23 spent relatively little time at the foraging site but visited it frequently. Some termites (e.g., individual #4, #24, and #29) visited the foraging site only once or twice for short periods and did not return to the foraging site after one or two foraging activities. However, most termites (e.g., #7, #17, #38, and others) persistently participated in the foraging tasks during observation.

**Fig. 3:**
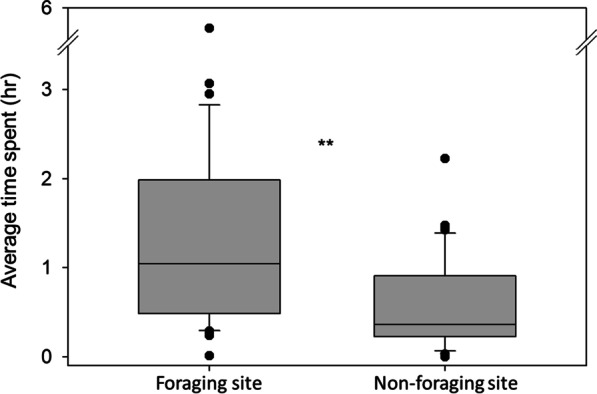
Box plot of time spent at the foraging site and non-foraging sites. A total of three colonies of *C. formosanus* were used, and 13 marked workers were observed per colony. Asterisk denote significant differences according to Mann–Whitney U test (*P* < 0.05)

Comparisons of foraging workers’ time allocation showed that workers, on average, spent significantly longer time at the foraging site than the non-foraging site (Mann–Whitney U test, *U* = 393.00, *P* < 0.01) (Fig. 3). Since the tendency of foraging workers’ time allocation could be varied across colonies, it was further tested. However, variation in foraging workers’ time allocation among colonies was not detected. All colonies showed similar patterns that foragers spent a significantly longer time at the foraging site (colony1: *U* = 42.00, *P* = 0.029; colony 2: *U* = 43.00, *P* = 0.034; colony 3: *U* = 42.00, *P* = 0.029) (Additional file 2: Figure 1).

### Two different types of foraging workers

**Fig. 4:**
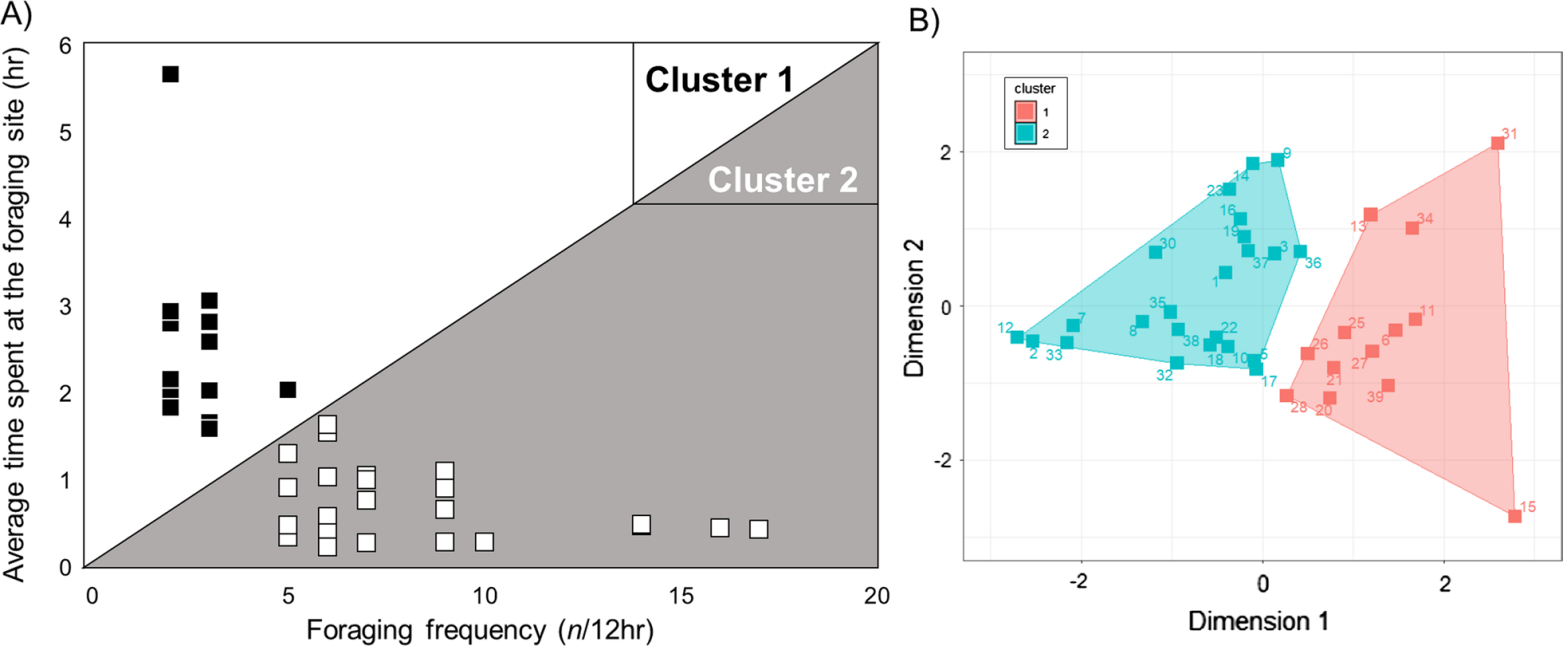
**A** Average time spent at the foraging site and foraging frequency of termites in two different clusters according to K-means clustering analysis. White and black squares indicate termites in clusters 1 and 2, respectively. **B** Results of K-means clustering analysis

Clustering analysis was performed to find out if foraging workers can be categorized by time spent at each location and foraging frequency. Two different clusters were found by K-means clustering analysis (Fig. 4), and these two clusters were further statistically differed in average time spent at the foraging site (*U* = 1.00, *P* < 0.01) and foraging frequency (*U* = 2.00, *P* < 0.01) (Additional file 3: Figure 2). However, time spent at the non-foraging site was not significantly different between the two clusters (*U* = 149.00, *P* = 0.987) (Additional file 3: Figure 2). Termites belonging to “cluster 1” spent, on average, longer time at the foraging site and showed less foraging frequency than termites in “cluster 2” (Fig. 3). Percentages of individuals belonging to each cluster were 25% (cluster 1) and 75% (cluster 2) in colony 1 and 42% (cluster 1) and 58% (cluster 2) in colonies 2 and 3.

**Fig. 5:**
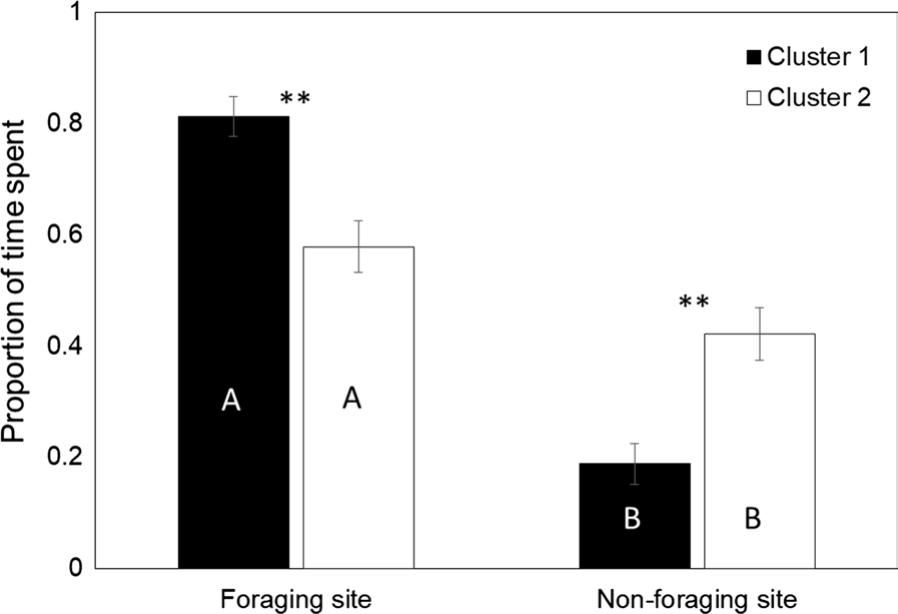
Proportion of time spent at the foraging and non-foraging sites by *C. formosanus* workers in clusters 1 (black) and 2 (white). Asterisks and upper case letters denote significant differences within and between clusters according to Wilcoxson sum rank test (*P* < 0.05), respectively

The proportion of time spent at the foraging site and non-foraging site was significantly different within clusters 1 and 2, and termites from both clusters spent significantly longer time at the foraging site than the non-foraging site (cluster 1: Wilcoxson sum rank test, *W* = 91.000, Fisher’s exact test, *P* < 0.01; cluster 2: *W* = 43.000, *P* = 0.020) (Fig. 5). A comparison between clusters showed that termites of cluster 1 spent proportionally more time at the foraging site than those of cluster 2 (*W* = 326.00, *P* < 0.01). Conversely, termites in cluster 2 spent proportionally longer time at the non-foraging site relative to termites in cluster 1 (*W* = 151.000, *P* < 0.01).

## Discussion

Foraging behaviors (e.g., food discovery, acquisition, and transportation) in social insects are collectively performed by individuals [37, 38], and various social insects have been investigated to find out how they forage. In contrast, studies on foraging behaviors in subterranean termites have primarily focused on tunneling behaviors (excavation and deposition of soil particles) to determine how foragers can optimize their chance of discovering wood resources while minimizing the inherent cost associated with tunneling activity [30, 39]. Such studies focused on food finding processes in subterranean termites because of their importance to understanding how subterranean termite pest species can find bait stations used in control application. Our study focused on differential time allocation among workers at a foraging site after a food source was located by tunneling workers. Our observations initially suggested that most of the marked workers continuously participated in foraging tasks, with some variation in the level of behavioral plasticity among individuals (Additional file 1: Table 1).

In this study, two different types of foraging workers were identified (Fig. 4). Both the duration of time spent at the foraging site and the foraging frequency of individuals were contributing factors for the clustering analysis, which indicated a behavioral dichotomy in foraging workers of subterranean termites. Workers that segregated in “cluster 1” remained at the foraging site for a relatively long time and performed few commuting events outside of the foraging site. Conversely, workers that segregated in “cluster 2” spent relatively short time at the foraging site, but had relatively high foraging frequencies (coming in-and-out of the foraging site). Some individuals showed an extreme display of these behaviors, either in food acquisition (#5, #15, #22, #28 and #39, Additional file 1: Table 1) or almost exclusively commuting in-and-out of the foraging site (#12, #22 and #33, Additional file 1: Table 1), supporting the presence of two different behavioral types in workers at foraging sites in *C. formosanus.*

Our results suggest that subterranean termites can be temporarily specialized in different aspects of foraging activity, such as food finding, acquisition, and transportation, to increase overall foraging efficiency similar to tunneling behaviors [20, 22, 23]. Food finding efficiency is determined by the geometry of underground tunnels, which are optimized to find resources with clumped distributions [30]. However, once tunnels are established between the nest and foraging sites, food finding efficiency may no longer be the most pressing priority for the colony, and workers that are temporarily the most fit to acquire wood can be recruited to the foraging site [40]. Hence, time allocation of foraging workers in food acquisition at the foraging site and transportation will become a more crucial factor to increase the overall foraging efficiency of the colony until they need to find a new food source.

However, it is currently unknown how workers become specialized in a certain task in termites. One possibility of such specialization in food acquisition behavior is that it may inherently be imposed by the physiological constraints of termite workers. Although the mandibles of termites are hardened with metal components [41], chewing on wood imposes considerable stress on worker mandibles, causing them to become dull over time [42, 43]. Therefore, individuals that recently molted and have sharp mandibles are likely to be more efficient at wood processing than workers that possess dull mandibles [44]. In leaf-cutting ants, task division in foraging by the level of mandible sharpness was observed and workers change tasks from cutters to transporters once their mandibles wear out, as it may take more time to cut leaves with dull mandibles [45]. Therefore it is possible that termite behavioral specialization in foraging may partially be driven by their hemimetabolous development. For instance, workers of *C. formosanus* molt every 45 days with a daily molting rate of about 2% [46]. In this scenario, subterranean termite colonies could have a wide range of workers that vary in terms of mandible sharpness ranging from very sharp after a recent molt and the most efficient in the food acquisition, to dull, which may not be efficient in the food processing [44]. Our observation of some foraging individuals only showing up at the foraging site briefly and for only a few times also suggests the possibility that workers with dull mandibles may stop foraging and go through molting process [47]. These hypotheses regarding changes in behavior according to the molting cycle remain to be fully tested, but our current observations suggests such possibilities.

Here, we provided preliminary evidence of food transportation specialization in workers. Some workers exited the foraging site toward the central nest with food boluses, but then returned to the foraging site after a short time without food visible in their mouthparts (Fig. 2), suggesting that it was transferred to other workers in the tunnel. One way to increase transportation efficiency is to reduce traffic in the tunnel, as heavy traffic in congested tunnels may negatively affect the movement of individuals. Previous studies reported that tunnel widths of *C. formosanus* ranged from 2 to 5 mm [48, 49]. Considering the narrow widths of underground tunnels between the nest and the foraging site, this will likely cause heavy traffic at the entrance of the foraging site if all foraging workers display high foraging frequencies. Therefore, commuting workers that carry food loads may avoid and minimize high traffic in the tunnel, which may ultimately increase foraging efficiency. In contrast, if all workers exhibit low foraging frequency, it is possible to generate a queuing delay at the foraging site, especially near food sources, which would also not favorable to maximize foraging efficiency. Therefore, task specialization of foraging workers, as inferred by their time allocation, might be driven not only by the temporary physiological status of any given worker, but also by a collective behavior to minimize the traffic.

Task specialization within foraging cohorts was previously reported in various social insects, including some termite species, but not in subterranean termites. In a harvester termite (open forager), *Hodotermes mossambicus* (Hagen) (Hodotermitidae), some workers in the foraging cohorts cut short grass pieces and deposit them along the foraging trail, where they are then later picked up by other workers, which eventually transport the food back to the nest [50]. In another open forager, *Macrotermes* (Termitidae, Macrotermitinae), foraging workers often process food at the foraging site and carry it to the nest following chemical trails [13]. However, it was observed that many workers take the food directly to the nest after foraging, while some workers can also display stomodeal trophallaxis to recipient workers. The recipient carries the food back to the central nest, while worker donors return to the foraging site after trophallaxis events [13]. The observation that foraging workers in Termitidae, Hodotermitidae, and now in Rhinotermitidae perform different tasks at the foraging site raises the question of if such task division in foraging is a basal trait in one of the common ancestors of these families, or if it evolved independently in these three taxa, as they all display a central nest structure with extended foraging sites [51]. Further studies are necessary to determine the potential driving forces of such polyethism in task division efficiency, not only in *Coptotermes*, but in other termite models with similar physiology and life traits.

## Supplementary Information

**Additional file 1: Table 1.** Individual tracking data of marked foraging workers in *C. formosanus*. In total, 13 termites per colony were measured and 3 different colonies were used (Colony 1: 1–13; colony 2: 14–26; colony 3: 27–39). Average and total time spent were calculated by averaging and summation all incidences respectively.

**Additional file 2: Figure 1.** Comparison of foraging workers’ average time spent at the foraging site (gray) and non-foraging site (blank) based on each colony.

**Additional file 3: Figure 2.** Average of time spent at the foraging site (A), at the non-foraging site (B) and foraging frequency (C) of cluster 1 and 2. Asterisk and NS denote significant and no differences respectively according to Mann-Whitney U test (P < 0.05).

## Data Availability

Data generated from this study is included in supplementary information.
