# Supplementary material for: Differential time allocation of foraging workers in the subterranean termite
Source: Front Zool. 2021 Dec 13;18:61. doi: 10.1186/s12983-021-00446-5 (PMC8670135; doi:10.1186/s12983-021-00446-5)
Supplement: Supplementary file 1 — Additional file 1: Table 1. Individual tracking data of marked foraging workers in C. formosanus. In total, 13 termites per colony were measured and 3 different colonies were used (Colony 1: 1–13; colony 2: 14–26; colony 3: 27–39). Average and total time spent were calculated by averaging and summation all incidences respectively. [file 12983_2021_446_MOESM1_ESM.docx]

Supplementary table 1. Individual tracking data of marked foraging workers in *C. formosanus*. In total, 13 termites per colony were measured and 3 different colonies were used (Colony 1: 1-13; colony 2: 14-26; colony 3:27-39). Average and total time spent were calculated by averaging and summation all incidences respectively.

|  | Termite ID | Foraging frequency (n) | Average time spent (hr) | | Total time spent (hr) | | Proportion of time spent | |
| --- | --- | --- | --- | --- | --- | --- | --- | --- |
| Site  Colony |  |  | Foraging site | Non-foraging site | Foraging site | Non-foraging site | Foraging site | Non-foraging site |
| Colony 1 | 1 | 7 | 0.77 | 0.74 | 5.42 | 5.21 | 0.51 | 0.49 |
|  | 2 | 16 | 0.46 | 0.20 | 7.34 | 2.99 | 0.71 | 0.29 |
|  | 3 | 5 | 0.92 | 0.91 | 4.62 | 4.56 | 0.50 | 0.50 |
|  | 4 | 1 | 1.02 | 0.01 | 1.02 | 0.01 | 0.99 | 0.01 |
|  | 5 | 6 | 1.56 | 0.27 | 9.35 | 1.60 | 0.85 | 0.15 |
|  | 6 | 3 | 2.83 | 0.79 | 8.48 | 1.58 | 0.84 | 0.16 |
|  | 7 | 14 | 0.48 | 0.31 | 6.78 | 4.08 | 0.62 | 0.38 |
|  | 8 | 9 | 0.30 | 0.27 | 2.66 | 2.41 | 0.52 | 0.48 |
|  | 9 | 5 | 0.36 | 1.48 | 1.78 | 5.90 | 0.23 | 0.77 |
|  | 10 | 6 | 1.04 | 0.25 | 6.26 | 1.24 | 0.83 | 0.17 |
|  | 11 | 2 | 2.81 | 0.86 | 5.62 | 1.73 | 0.77 | 0.23 |
|  | 12 | 17 | 0.44 | 0.23 | 7.51 | 3.68 | 0.67 | 0.33 |
|  | 13 | 3 | 1.67 | 1.38 | 5.02 | 2.76 | 0.65 | 0.35 |
| Colony 2 | 14 | 6 | 0.24 | 1.43 | 1.44 | 7.13 | 0.17 | 0.83 |
|  | 15 | 2 | 5.67 | 0.09 | 11.35 | 0.09 | 0.99 | 0.01 |
|  | 16 | 6 | 0.40 | 1.05 | 2.43 | 5.25 | 0.32 | 0.68 |
|  | 17 | 6 | 1.64 | 0.22 | 9.86 | 1.12 | 0.90 | 0.10 |
|  | 18 | 7 | 1.01 | 0.25 | 7.10 | 1.78 | 0.80 | 0.20 |
|  | 19 | 6 | 0.59 | 0.96 | 3.52 | 5.77 | 0.38 | 0.62 |
|  | 20 | 2 | 1.99 | 0.07 | 3.98 | 0.13 | 0.97 | 0.03 |
|  | 21 | 2 | 1.84 | 0.26 | 3.68 | 0.53 | 0.87 | 0.13 |
|  | 22 | 7 | 1.06 | 0.33 | 7.41 | 1.97 | 0.79 | 0.21 |
|  | 23 | 7 | 0.29 | 1.25 | 2.01 | 8.77 | 0.19 | 0.81 |
|  | 24 | 2 | 0.01 | 0.03 | 0.02 | 0.03 | 0.41 | 0.59 |
|  | 25 | 3 | 2.03 | 0.58 | 6.10 | 1.74 | 0.78 | 0.22 |
|  | 26 | 3 | 1.60 | 0.31 | 4.79 | 0.94 | 0.84 | 0.16 |
| Colony 3 | 27 | 3 | 2.60 | 0.58 | 7.79 | 1.73 | 0.82 | 0.18 |
|  | 28 | 5 | 2.04 | 0.12 | 10.21 | 0.60 | 0.94 | 0.06 |
|  | 29 | 1 | 1.30 | 1.00 | 1.30 | 1.00 | 0.57 | 0.43 |
|  | 30 | 10 | 0.29 | 0.79 | 2.95 | 7.13 | 0.29 | 0.71 |
|  | 31 | 2 | 2.95 | 2.23 | 5.90 | 4.45 | 0.57 | 0.43 |
|  | 32 | 9 | 1.11 | 0.15 | 10.00 | 1.24 | 0.89 | 0.11 |
|  | 33 | 14 | 0.50 | 0.19 | 6.99 | 2.60 | 0.73 | 0.27 |
|  | 34 | 2 | 2.17 | 1.39 | 4.33 | 2.78 | 0.61 | 0.39 |
|  | 35 | 9 | 0.67 | 0.43 | 6.00 | 3.46 | 0.63 | 0.37 |
|  | 36 | 5 | 1.31 | 1.02 | 6.57 | 4.10 | 0.62 | 0.38 |
|  | 37 | 5 | 0.49 | 0.83 | 2.44 | 3.30 | 0.43 | 0.57 |
|  | 38 | 9 | 0.91 | 0.36 | 8.20 | 3.24 | 0.72 | 0.28 |
|  | 39 | 3 | 3.07 | 0.44 | 9.20 | 0.87 | 0.91 | 0.09 |
