# Supplementary figures and images for: Differential time allocation of foraging workers in the subterranean termite
Source: Front Zool. 2021 Dec 13;18:61. doi: 10.1186/s12983-021-00446-5 (PMC8670135; doi:10.1186/s12983-021-00446-5)

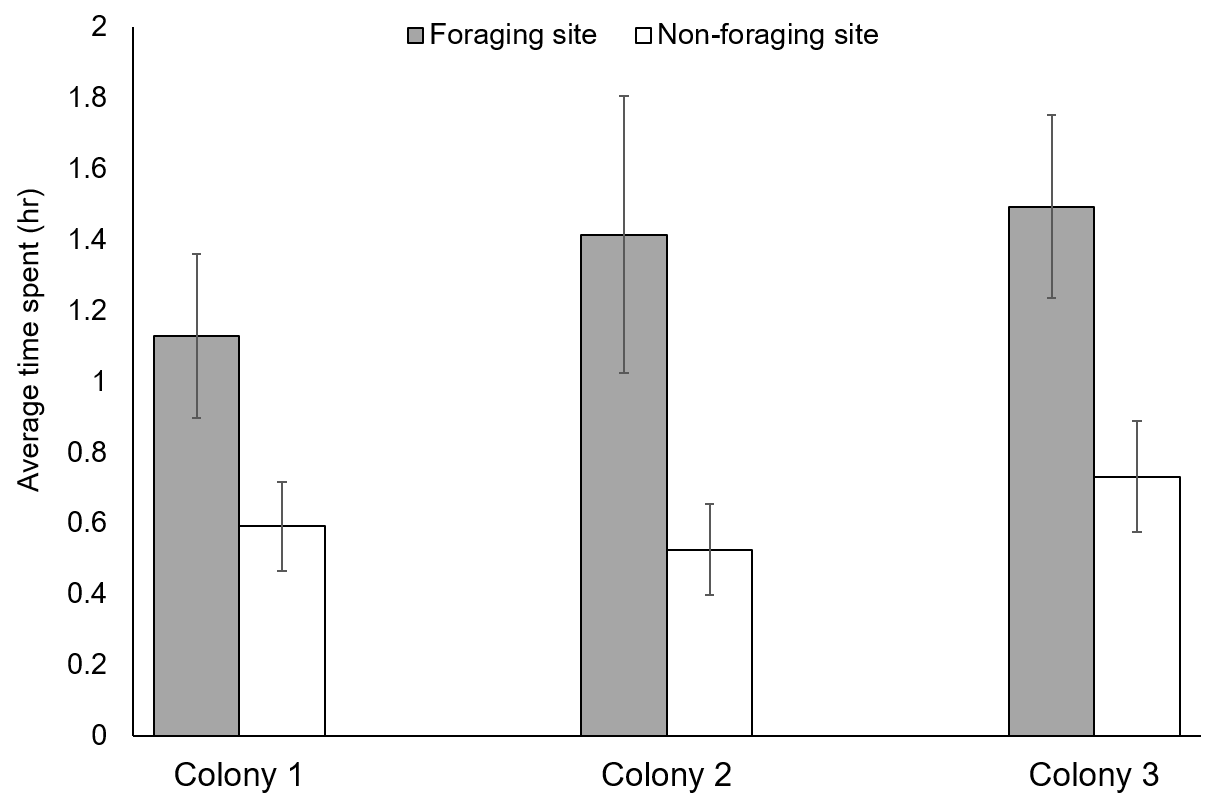

Supplement: Supplementary file 2 — Additional file 2: Figure 1. Comparison of foraging workers’ average time spent at the foraging site (gray) and non-foraging site (blank) based on each colony. [file 12983_2021_446_MOESM2_ESM.tif]

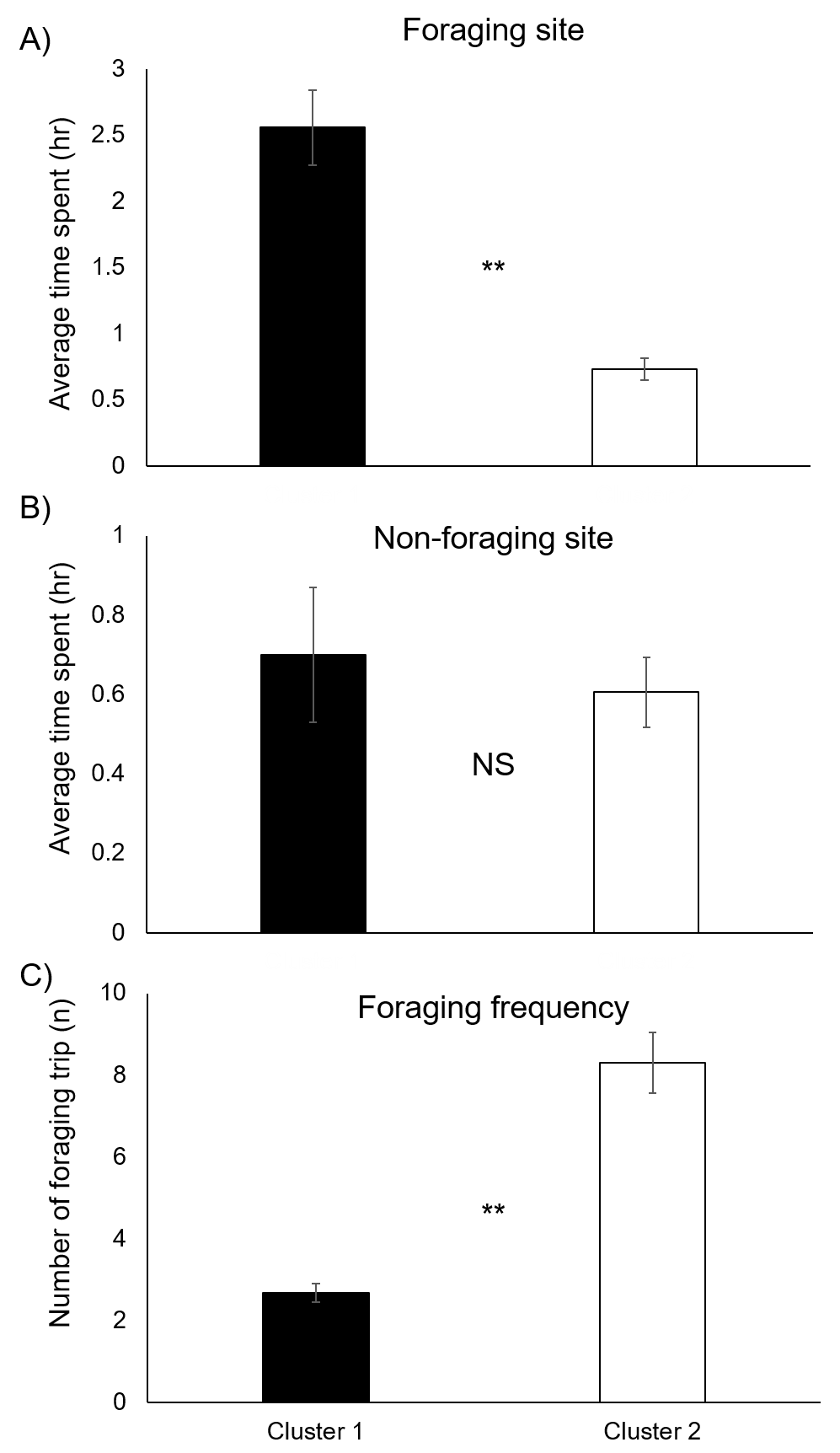

Supplement: Supplementary file 3 — Additional file 3: Figure 2. Average of time spent at the foraging site (A), at the non-foraging site (B) and foraging frequency (C) of cluster 1 and 2. Asterisk and NS denote significant and no differences respectively according to Mann-Whitney U test (P < 0.05). [file 12983_2021_446_MOESM3_ESM.tif]
